# Bias induced up to 100% spin-injection and detection polarizations in ferromagnet/bilayer-hBN/graphene/hBN heterostructures

**DOI:** 10.1038/s41467-017-00317-w

**Published:** 2017-08-15

**Authors:** M. Gurram, S. Omar, B. J. van Wees

**Affiliations:** Physics of Nanodevices, Zernike Institute for Advanced Materials, University of Groningen, Nijenborgh 4, Groningen, 9747 AG, The Netherlands

## Abstract

We study spin transport in a fully hBN encapsulated monolayer-graphene van der Waals heterostructure at room temperature. A top-layer of bilayer-hBN is used as a tunnel barrier for spin-injection and detection in graphene with ferromagnetic cobalt electrodes. We report surprisingly large and bias-induced (differential) spin-injection (detection) polarizations up to 50% (135%) at a positive voltage bias of + 0.6 V, as well as sign inverted polarizations up to −70% (−60%) at a reverse bias of −0.4 V. This demonstrates the potential of bilayer-hBN tunnel barriers for practical graphene spintronics applications. With such enhanced spin-injection and detection polarizations, we report a record two-terminal (inverted) spin-valve signals up to 800 Ω with a magnetoresistance ratio of 2.7%, and achieve spin accumulations up to 4.1 meV. We propose how these numbers can be increased further, for future technologically relevant graphene based spintronic devices.

## Abstract (web summary)

In 2D spin-valve devices, effective spin injection and detection can be potentially realised combining graphene with an ideal hBN tunnel barrier. Here, the authors report that a bilayer hBN tunnel barrier allows up to 100% spin-injection and detection in a fully hBN-encapsulated graphene heterostructure.

## Introduction

Recent progress in the exploration of various two-dimensional materials has led to special attention for van der Waals (vdW) heterostructures for advanced graphene spintronics devices. For graphene spin-valve devices, an effective injection and detection of spin-polarized currents with a ferromagnetic (FM) metal via efficient tunnel barriers is crucial^1, 2^. The promising nature of crystalline hexagonal boron nitride (hBN) layers as pin-hole free tunnel barriers^3^ for spin injection into graphene^4–8^ has been recently demonstrated. However, due to the relatively low interface resistance-area product of monolayer-hBN barriers, there is a need to use a higher number of hBN layers for non-invasive spin injection and detection^9^. Theoretically, large spin-injection polarizations have been predicted in FM/hBN/graphene systems as a function of bias with increasing number of hBN layers^10^.

Kamalakar et al.^11^ reported an inversion of the spin-injection polarization for different thicknesses of chemical vapour deposited (CVD)-hBN tunnel barriers, as well as an asymmetric bias dependence of the polarization using multilayer CVD-hBN/FM tunnel contacts. The observed behavior was attributed to spin-filtering processes across the graphene/multilayer-hBN/FM tunnel contacts.

In order to explore the potential of hBN tunnel barriers for graphene spin valve devices, one can study the role of current/voltage bias for spin-injection and detection with FM electrodes. Application of a bias across the FM/hBN/graphene tunneling contacts (a) allows to widen the energy window up to ~1 eV for additional spin polarized states in the FM and graphene to participate in the tunneling spin-injection and detection processes, (b) induces a large electric-field between the FM and graphene, which can modify the tunneling processes, (c) provides electrostatic gating for the graphene, which could change the carrier density between electrons and holes, and (d) is predicted to induce magnetic proximity exchange splitting in graphene of up to 20 meV^12, 13^.

Here we show that bilayer(2L)-hBN tunnel barriers are unique for spin-injection and detection in graphene, with (differential) polarizations unexpectedly reaching values close to ±100% as a function of the applied DC bias at room temperature. Furthermore, we demonstrate a two-terminal (inverted) spin-valve with a record magnitude of the spin signal reaching 800 Ω with magnetoresistance ratio of 2.7%.

## Results

### Four-terminal non-local spin transport

**Fig. 1:**
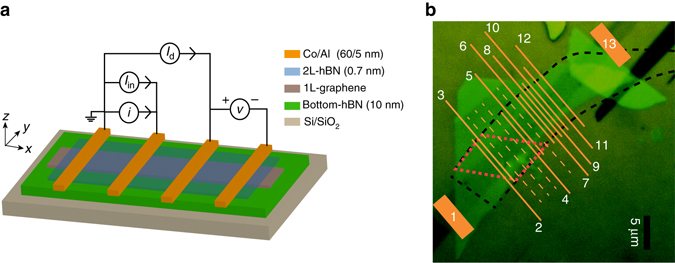
Device layout and measurement scheme. **a** A layer-by-layer schematic of the vdW heterostructure of the 2L-hBN/graphene/thick-hBN stack with FM cobalt electrodes. A measurement scheme is shown for the non-local spin transport measurements with a DC current bias *I*_in_ and AC current *i*, applied across the injector contacts and a non-local differential (AC) spin signal *v* is measured using a lock-in detection technique. A DC current bias *I*_d_ can also be applied in order to bias the detector contact. **b** An optical microscopic picture of the vdW heterostructure. *Scale bar*, 5 μm. The *black-dashed line* outlines the hBN tunnel barrier flake. The *red-dashed line* outlines the monolayer region of the hBN tunnel barrier flake (see Supplementary Note 1 for the optical microscopic picture of the tunnel barrier). A schematic of the deposited cobalt electrodes is shown as *orange bars* and the Co/hBN/graphene contacts are denoted by numbers 1, 2, .., and 13. The *orange-dashed lines* represent the unused contacts. Cobalt electrodes from 2 to 5 are either fully or partially deposited on top of the monolayer region of the tunnel-barrier flake, while the electrodes from 6 to 12 are exclusively deposited on the bilayer region. The width of the cobalt electrodes (2–12) is varied between 0.15 and 0.4 μm

**Fig. 2:**
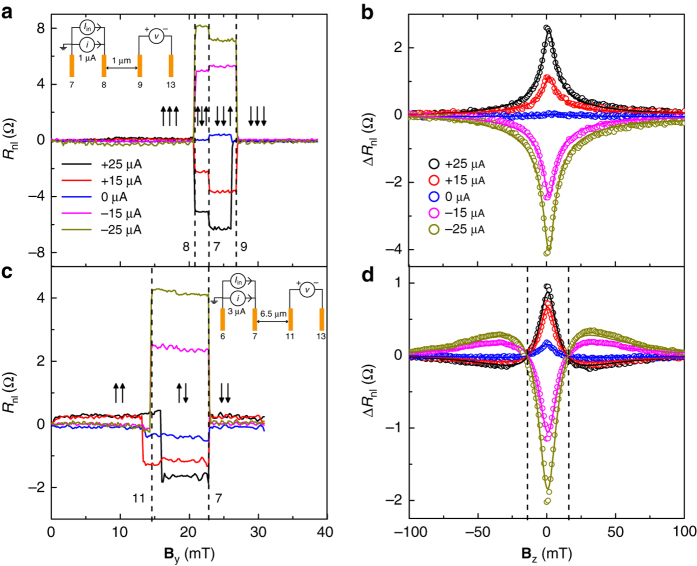
Non-local spin-valve and Hanle measurements at different DC bias across the injector. **a**, **c**: Non-local differential spin-valve signal *R*_nl_ (=*v*/*i*) as a function of the magnetic field **B**_y_ applied along the easy axes of the Co electrodes, for a short (*L* = 1 μm) **a**, and a long (*L* = 6.5 μm) **c** spin transport channel. An offset at zero field is subtracted from each curve for a clear representation of the data. The *vertical dashed lines* correspond to the switching of the electrodes at their respective coercive fields. The switch of the outer detector 13 is not detectable as it is located far (>2*λ*_s_) from the nearest injector. The legend shows the applied injection DC current bias *I*_in_ values. The *up* (↑) and *down* (↓) *arrows* represent the relative orientation of the electrode magnetizations. The three *arrows* in **a** correspond to the contacts 7, 8, and 9, and the two *arrows* in **c** correspond to the contacts 7 and 11, from *left* to *right*. The *insets* in **a** and **c** show the measurements schematics, injection AC current (*i*) and the DC current bias (*I*_in_), the respective contacts used for the spin current injection, and non-local differential voltage (*v*) detection. The differential spin signal in **a** due to spin injection through 8 is $\Delta R_{{\rm{nl}}}^{{\rm{8}} - {\rm{9}}} = \left( {R_{{\rm{nl}}}^{ \uparrow \uparrow \uparrow } - R_{{\rm{nl}}}^{ \uparrow \downarrow \uparrow }} \right){\rm{/}}2$, and in **c** due to spin injection through 7 is $\Delta R_{{\rm{nl}}}^{{\rm{7}} - {\rm{11}}} = \left( {R_{{\rm{nl}}}^{ \uparrow \uparrow } - R_{{\rm{nl}}}^{ \uparrow \downarrow }} \right){\rm{/}}2$. **b**, **d** Non-local (differential) Hanle signal Δ*R*_nl_(**B**_z_) as a function of the magnetic field **B**_z_. **b**, **d** shows Δ*R*_nl_ measured for the short(long) channel, corresponding to the spin injector contact 8(7) and measured with the detector contact 9(11). The measured data are represented in circles and the solid lines represent the fits to the data. Hanle signals in **b** at different injection bias values $\Delta R_{{\rm{nl}}}^{{\rm{8}} - {\rm{9}}}\left( {{{\bf{B}}_{\rm{z}}}} \right) = \left( {R_{{\rm{nl}}}^{ \uparrow \uparrow \uparrow }\left( {{{\bf{B}}_{\rm{z}}}} \right) - R_{{\rm{nl}}}^{ \uparrow \downarrow \uparrow }\left( {{{\bf{B}}_{\rm{z}}}} \right)} \right){\rm{/}}2$. The two *vertical dashed lines* in **d** correspond to the fields where the Hanle signals cross zero

**Fig. 3:**
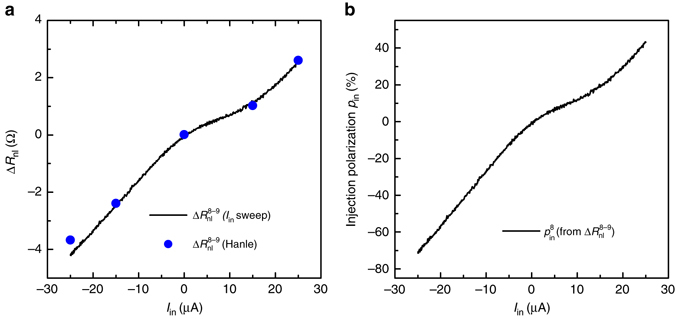
Bias enhanced non-local differential spin signal and large differential spin-injection polarization at room temperature. **a** Non-local spin signal $\Delta R_{{\rm{nl}}}^{{\rm{8}} - {\rm{9}}}$(*I*_in_) corresponding to the spin current injected through contact 8 and detected via contact 9, as a function of the DC current bias (*I*_in_) applied across the injector. The *solid line* represents the spin signal $\Delta R_{{\rm{nl}}}^{{\rm{8}} - {\rm{9}}}$(*I*_in_) for a continuous sweeping of the *I*_in_ bias, while the *dots* are extracted from the Hanle signals $\Delta R_{{\rm{nl}}}^{{\rm{8}} - {\rm{9}}}$(**B**_z_) at **B**_*z*_ = 0, measured at different bias (from Fig. 2b). **b** Differential spin-injection polarization of the injector contact 8, $p_{{\rm{in}}}^{\rm{8}}$ as a function of *I*_in_, calculated from the $\Delta R_{{\rm{nl}}}^{{\rm{8}} - {\rm{9}}}$(*I*_in_) (Eq. (3)) data plotted in **a**

We study the spin transport in fully hBN-encapsulated graphene, prepared via dry pick-up and transfer method^14^ to obtain clean and polymer free graphene-hBN interfaces^4^ (see “Methods” section for device fabrication details). We use a four-terminal non-local measurement geometry to separate the spin current path from the charge current path (Fig. 1a). An AC current (*i*) is applied between two Co/2L-hBN/graphene contacts to inject a spin-polarized current in graphene. The injected spin accumulation in graphene diffuses and is detected non-locally (*v*) between the detector contacts using a low-frequency (*f* = 10–20 Hz) lock-in technique. For the spin-valve measurements, the magnetization of all the contacts is first aligned by applying a magnetic field **B**_y_ along their easy axes. Then **B**_y_ is swept in the opposite direction. The magnetization reversal of each electrode at their respective coercive fields appears as an abrupt change in the non-local differential resistance *R*_nl_ (=*v*/*i*). Along with a fixed amplitude *i* of 1–3 μA, we source a DC current (*I*_in_) to vary the bias applied across the injector contacts. In this way, we can obtain the differential spin-injection polarization of a contact, defined as ${p_{{\rm{in}}}} = \frac{{{i_{\rm{s}}}}}{i} = \frac{{{\rm{d}}{I_{\rm{s}}}}}{{{\rm{d}}I}}$, where *I*_s_ (*i*_s_) are the DC (AC) spin currents, and study in detail how *p*_in_ of the contacts depends on the applied bias. We observe with bilayer-hBN tunnel barrier that the magnitude of the differential spin signal Δ*R*_nl_ at a fixed AC injection current increases with the DC bias applied across the injector (Fig. 2a, c). Moreover, a continuous change in the magnitude of Δ*R*_nl_ between −4.5 and 2.5 Ω as a function of DC current bias across the injector, and its sign reversal close to zero bias can be clearly observed (Fig. 3a). A similar behavior is also observed for different injection contacts.

In Hanle spin-precession measurements, where the magnetic field **B**_z_ is swept perpendicular to the plane of spin injection, the injected spins precess around the applied field and dephase while diffusing towards the detectors. We obtain the spin transport parameters such as spin-relaxation time *τ*_s_ and spin-diffusion constant *D*_s_ by fitting the non-local Hanle signal Δ*R*_nl_(**B**_z_) with the stationary solutions to the steady state Bloch equation in the diffusion regime; *D*_s_ *∇* ^2^ **μ**_s_ − **μ**_s_/*τ*_s_ + γ**B**_z_ × **μ**_s_ = 0. Here, the net spin accumulation *μ*_s_ is the splitting of spin chemical potentials spin-up *μ*_↑_ and spin-down *μ*_↓_, i.e., (*μ*_↑_ − *μ*_↓_)/2, and γ is the gyromagnetic ratio. In order to obtain reliable fitting parameters, we probe the Hanle signals for a long spin transport channel of length *L* = 6.5 μm. We measure the Hanle signals for different DC current bias and obtain the fitting parameters *τ*_s_ ~ 0.9 ns, *D*_s_ ~ 0.04 m^2^ s^−1^, and *λ*_s_ ~ 5.8 μm. We estimate the carrier density $n \simeq 5 \times {10^{12}}$ cm^−2^ from the Einstein relation and the carrier mobility *μ* ~ 3000 cm^2^ V^−1^ s^−1^ form the Drude’s formula, by assuming *D*_s_ = *D*_c_^15^, where *D*_c_ is the charge-diffusion constant. Both the mobility and spin relaxation time are relatively low, which could be due to the ineffective screening of the very thin (~0.7 nm) top-layer of bilayer-hBN from the contamination on the top surface. Due to non-functioning backgate of the device, we could not measure the carrier density dependence of these parameters. For the calculation of mobility, see Supplementary Note 8.

### Spin-injection polarization

Since *λ*_s_ does not change due to the bias applied between the injector contacts, the bias dependence of the non-local differential spin signal Δ*R*_nl_ in Figs. 2, 3a is due to the change in spin-injection polarization. From Δ*R*_nl_ in Fig. 3a, we can obtain the differential spin-injection polarization of the injector contact 8, $p_{{\rm{in}}}^8$ from ref. ^16^.

1 $$\Delta R_{{\rm{nl}}}^{8 - 9} = \frac{{{R_{{\rm{sq}}}}{\lambda _{\rm{s}}}}}{{2W}}\left[ {p_{{\rm{in}}}^8p_{\rm{d}}^9{e^{\frac{{ - L}}{{{\lambda _{\rm{s}}}}}}}} \right],$$

using a known unbiased detection polarization of detector 9, $p_{\rm{d}}^9$ (see Supplementary Note 3 for the analysis and calculation of $p_{\rm{d}}^9$), the length between contacts 8 and 9, *L*_8−9_ = 1 μm, the square resistance *R*_sq_ ~ 400 Ω, and the width *W* = 3 μm of graphene. The non-local spin signal as a function of bias due to the spin injection through 8 is obtained from $\Delta R_{{\rm{nl}}}^{{\rm{8}} - {\rm{9}}}\left( {{I_{{\rm{in}}}}} \right) = \left( {R_{{\rm{nl}}}^{ \uparrow \uparrow \uparrow }\left( {{I_{{\rm{in}}}}} \right) - R_{{\rm{nl}}}^{ \uparrow \downarrow \uparrow }\left( {{I_{{\rm{in}}}}} \right)} \right){\rm{/}}2$, where $R_{{\rm{nl}}}^{ \uparrow \uparrow \uparrow }\left( {{I_{{\rm{in}}}}} \right)$ is the non-local signal measured as a function of *I*_in_ when the magnetization of contacts 7, 8, and 9 are aligned in ↑, ↑, and ↑ configuration, respectively. We find that $p_{{\rm{in}}}^8$ changes from −1.2% at zero bias to +40% at +25 μA and −70% at −25 μA (Fig. 3b). It shows a sign inversion, which occurs close to zero bias. The absolute sign of *p* cannot be obtained from the spin transport measurements and we define it to be positive for the majority of the unbiased contacts (Supplementary Note 3).

The observed behavior of the (differential) polarization is dramatically different from what has been observed so far for spin-injection in graphene, or in any other non-magnetic material. For spin-injection/detection with conventional FM tunnel contacts, the polarization does not change its sign close to zero bias. It can be modified at high bias^17^. However, in our case, we start with a very low polarization at zero bias which can be enhanced dramatically in positive and negative directions.

**Fig. 4:**
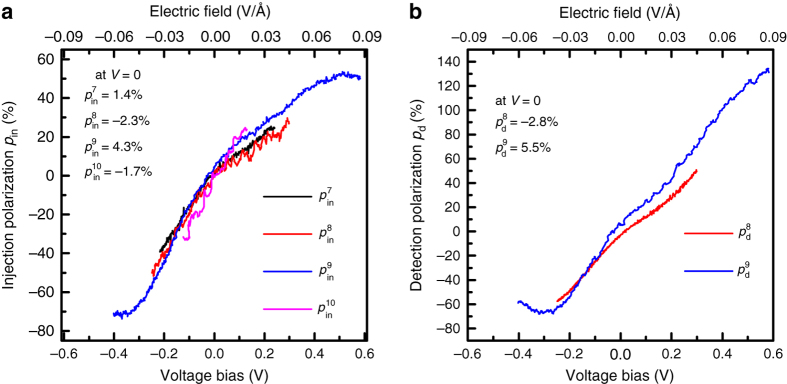
Differential spin-injection (*p*_in_) and detection (*p*_d_) polarizations of the cobalt/bilayer-hBN/graphene contacts. **a** Differential spin-injection polarization *p*_in_ of four contacts with 2L-hBN tunnel barrier, as a function of the DC voltage bias *V*. *Top axis* represents the corresponding electric-field (=*V*/*t*_hBN_, *t*_hBN_ ≈7 Å, the thickness of 2L-hBN barrier) induced across the Co/2L-hBN/graphene contacts. Note that the Δ*R*_nl_ used to calculate $p_{{\rm{in}}}^8$ in Fig. 3b is obtained from a different data set. **b** Differential spin-detection polarization *p*_d_ of contacts 8 and 9 as a function of DC voltage bias *V* applied across the detector, while the injector bias is fixed at *I*_in_ = + 20 μA. The *insets* in **a** and **b** show *p*_in_ and *p*_d_ of contacts at zero bias, respectively. The *top x axis* in **a** and **b** indicates the electric field corresponding to the applied bias across the contacts

The above analysis is repeated for other bilayer-hBN tunnel barrier contacts with different interface resistances. Figure 4a shows *p*_in_ for four contacts plotted as a function of the voltage bias obtained from the respective Δ*R*_nl_(*I*_in_). All contacts show similar behaviour, where the magnitude of *p*_in_ increases with bias and changes sign close to zero bias. For the same range of the applied voltage bias, contacts with either 1L-hBN or TiO_2_ tunnel barriers do not show a significant change in the spin polarization (Supplementary Notes 6, 10). This behavior implies that the observed tunneling spin-injection polarization as a function of the bias is unique to bilayer-hBN tunneling contacts.

### Spin-detection polarization

We now study the effect of the bias on spin-detection. The (differential) spin detection polarization *p*_d_ of a contact is defined as the voltage change (Δ*V*) measured at the detector due to a change in the spin accumulation underneath (Δ*μ*_s_) (see Supplementary Note 2 for the derivation and details),

2 $${p_{\rm{d}}} = \frac{{\Delta V}}{{\Delta {\mu _{\rm{s}}}{\rm{/}}e}}$$

where $\Delta V = i\left[ {\Delta R_{{\rm{nl}}}^{{\rm{in}} - {\rm d}}}\left( {{I_{{\rm d}}}} \right) \right]$ is measured as a function of the detector bias *I*_d_, and $\Delta {\mu _{\rm{s}}}{\rm{/}}e = \frac{{i{R_{{\rm{sq}}}}{\lambda _{\rm{s}}}}}{{2W}}{p_{{\rm{in}}}}{{\rm e}^{ - L/{\lambda _{\rm{s}}}}}$. In a linear response regime at low bias, *p*_d_ should resemble *p*_in_ because of reciprocity. However, in the non-linear regime at higher bias, they can be different. A comparison between Fig. 4a and b shows that the bias dependence of *p*_in_ and *p*_d_ is similar (see Supplementary Note 4 for determining *p*_d_ as a function of bias). However, we find that *p*_d_ of contact 9 can reach more than 100% above +0.4 V (corresponding electric field is +0.06 V Å^−1^). We note that the presence of a non-zero DC current in the graphene spin transport channel between injector and detector could modify *λ*_s_ due to carrier drift, and consequently the calculated polarizations have a typical uncertainty of about 10% (Supplementary Note 9). Although there is no fundamental reason that the biased detection polarizations *p*_d_ cannot exceed 100% (Supplementary Note 2), it could be that our observation of over 100% polarization is due to effect of the drift which is expected to have a bigger effect on the accurate determination of *p*_d_(*I*) as compared to *p*_in_(*I*) (Fig. 1a). Due to heating effect at the injector, there is also a possibility of thermal spin injection, which might result in an enhanced contact polarization. We make a rough estimate for this effect and find that the thermal effects due to large values of DC current are negligible on the spin transport as explained in Supplementary Note 7. We also verify the consistency of our approach from the calculation of DC spin injection polarization as shown in Supplementary Note 5.

Concluding, we have obtained a dramatic bias-induced increase in both the differential spin-injection and detection polarizations, reaching values close to ±100% as a function of applied bias across the cobalt/bilayer-hBN/graphene contacts.

### Two-terminal local spin transport

**Fig. 5:**
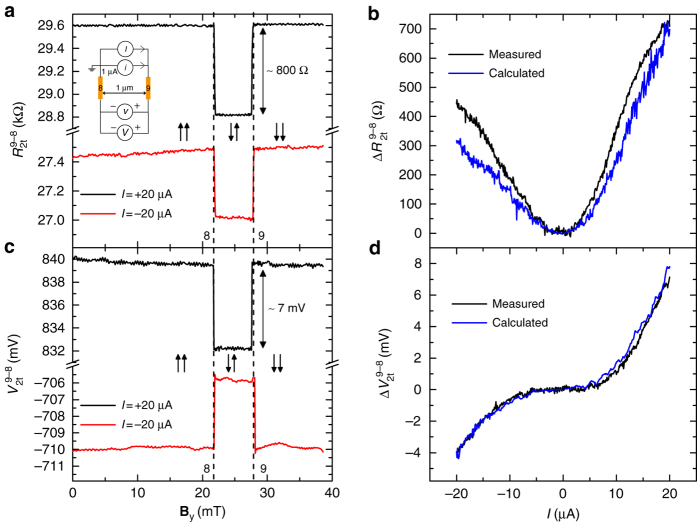
Large inverted two-terminal spin-valve effect at room temperature. **a** Two-terminal differential spin-valve signal *R*_2t_(=*v*/*i*) and **c** two-terminal DC spin-valve signal *V*_2t_, as a function of **B**_y_ at two different DC current bias values. The *inset* in **a** illustrates the two-terminal spin-valve measurement configuration. The *arrows* ↑↑ (↓↑) represent the parallel (anti-parallel) orientation of the magnetization of contacts 8 and 9, respectively, from *left* to *right*. The *vertical dashed lines* represent the coercive fields of contacts 8 and 9. **b** Two-terminal differential spin signal Δ*R*_2t_(*I*), and **d** two-terminal DC spin signal Δ*V*_2t_(*I*), as a function of the DC current bias *I*. The calculated two-terminal spin signals from the individual spin-injection and detection polarizations of contacts 8 and 9 are also shown in **b** and **d**

A four-terminal non-local spin-valve scheme is ideal for proof of concept studies, but it is not suitable for practical applications where a two-terminal local geometry is technologically more relevant. In a typical two-terminal spin-valve measurement configuration, the spin signal is superimposed on a (large) spin-independent background. Since we have found that the injection and detection polarizations of the contacts can be enhanced with DC bias, the two-terminal spin signal can now be large enough to be of practical use. For the two-terminal spin-valve measurements, a current bias (*i* + *I*) is sourced between contacts 8 and 9, and a spin signal (differential, *v* and DC, *V*) is measured across the same pair of contacts as a function of **B**_y_ (inset, Fig. 5a). Figure 5a, c shows the two-terminal differential resistance *R*_2*t*_ (=*v*/*i*) and the two-terminal DC voltage *V*_2*t*_, respectively, measured as a function of **B**_y_. As a result of the two-terminal circuit, both the contacts are biased with same *I* but with opposite polarity, resulting in opposite sign for the injection and detection polarizations. Therefore, we measure an inverted two-terminal differential spin-valve signal *R*_2t_ with minimum resistance in anti-parallel configuration. We observe a maximum magnitude of change in the two-terminal differential (DC) signal Δ*R*_2t_ (Δ*V*_2*t*_) of about 800 Ω (7 mV) at *I* = +20 μA, where $\Delta {R_{2{\rm{t}}}}\left( I \right) = R_{{\rm{2t}}}^{ \uparrow \uparrow }\left( I \right) - R_{2{\rm{t}}}^{ \downarrow \uparrow }\left( I \right)$ and $\Delta {V_{2{\rm{t}}}}\left( I \right) = V_{{\rm{2t}}}^{ \uparrow \uparrow }\left( I \right) - V_{{\rm{2t}}}^{ \downarrow \uparrow }\left( I \right)$ represent the difference in the two-terminal signals when the magnetization configuration of contacts 8 and 9 changes between parallel(↑↑) and anti-parallel (↓↑). A continuous change in Δ*R*_2t_ and Δ*V*_2t_ can be observed as a function of DC current bias (Fig. 5b, d).

The magnetoresistance (MR) ratio of the two-terminal differential spin signal is a measure of the local spin-valve effect, and is defined as $\left( {R_{{\rm{2t}}}^{ \downarrow \uparrow } - R_{{\rm{2t}}}^{ \uparrow \uparrow }} \right){\rm{/}}R_{{\rm{2t}}}^{ \uparrow \uparrow }$, where $R_{{\rm{2t}}}^{ \uparrow \downarrow }$ $\left( {R_{{\rm{2t}}}^{ \uparrow \uparrow }} \right)$ is the two-terminal differential resistance measured in the anti-parallel (parallel) magnetization orientation of the contacts. From the spin-valve signal, we calculate the maximum MR ratio of −2.7% at *I* = +20 μA.

Since we have already obtained the differential spin-injection and detection polarizations of both the contacts 8 and 9 as a function of bias (Fig. 4), we can calculate the two-terminal differential spin signal from

3 $$\Delta {R_{2{\rm{t}}}}(I) = \left[ {p_{{\rm{in}}}^9\left( {{I_{{\rm{in}}}}} \right)p_{\rm{d}}^8\left( { - {I_{\rm{d}}}} \right) + p_{{\rm{in}}}^8\left( { - {I_{{\rm{in}}}}} \right)p_{\rm{d}}^9\left( {{I_{\rm{d}}}} \right)} \right]\frac{{{R_{{\rm{sq}}}}{\lambda _{\rm{s}}}}}{W}{e^{ - \frac{L}{{{\lambda _{\rm{s}}}}}}}$$

The calculated differential signal Δ*R*_2t_(*I*) is plotted in Fig. 5b. A similar analysis can be done for the two-terminal DC spin signal Δ*V*_2t_(*I*) (Supplementary Note 2) and is plotted in Fig. 5d. Even though there is an uncertainty in the calculation of *p*_d_ due to a possible effect of carrier drift between the injector and detector, we get a close agreement between the measured and calculated signals in different (local and non-local) geometries. This confirms the accurate determination of the individual spin-injection and detection polarizations of the contacts.

Furthermore, we can now calculate the total spin accumulation in graphene, underneath each contact in the two-terminal biased scheme, due to spin-valve effect. The results are summarized in Table 1. The maximum spin accumulation, beneath contact 9, due to spin-injection/extraction from contacts 8 and 9 reaches up to 4.1 meV for an applied bias of *I* = +20 μA. It is noteworthy that such a large magnitude of spin accumulation in graphene at room temperature has not been reported before.

**Table 1:** Large spin accumulation underneath the contacts

	*μ*_s_ underneath 8 (meV)		*μ*_s_ underneath 9 (meV)	
	↑↑	↓↑	↑↑	↓↑
Injected by 8	1.8	−1.8	1.6	−1.6
Injected by 9	2.1	2.1	2.5	2.5
Total *μ*_s_	**3.9**	**0.3**	**4.1**	**0.9**

Spin accumulation *μ*_s_ in graphene, beneath the contacts, in the two-terminal spin-valve geometry at bias *I* = +20 μA. The arrows ↑↑ (↓↑) represent the parallel (anti-parallel) orientation of the magnetization of contacts 8 and 9, respectively, from left to right. Bold values represent the total spin accumulation in different magnetization orientation of contacts

## Discussion

Recent first-principles calculations of the proximity exchange coupling induced in graphene by Zollner et al.^12^ have predicted that an applied electric field in Co/hBN/graphene system can reverse the sign of the proximity-effect-induced equilibrium spin polarization in graphene (shown specifically for the case of 2L-hBN). Although this study is related to our experimental geometry, the exchange interactions are not relevant for the current discussion because we do not observe any signature of (bias-induced) exchange coupling on the shape of the Hanle signals (such as, as observed by Leutenantsmeyer et al.^18^ and Singh et al.^19^) except for the magnitude of the spin signals. Another study by Lazić et al.^13^ on the tunable proximity effects in Co/hBN/graphene has predicted that the system can be effectively gated, and both the magnitude and the sign of the equilibrium spin polarization of the density of states at the Fermi level can be changed due to transverse electric field. However, the spin polarization of the injected current is not calculated. Although these results are relevant to our study, further research is required to understand the results.

According to the first-principles transport calculations^10^, the bias-dependent spin-current injection efficiency from Ni into graphene increases up to 100% with the number of hBN tunnel barrier layers. However, these calculations do not show any sign inversion of the spin injection efficiency and do not predict any special role of bilayer-hBN. Therefore, we will not speculate further here on possible explanations of our fully unconventional observations. We note, however, that further research will require the detailed study of the injection/detection processes as a function of graphene carrier density, in particular, the interaction between contact bias induced and backgate induced carrier density. Via these measurements, one could also search for possible signatures of the recently proposed magnetic proximity exchange splitting in graphene with an insulator spacer, hBN^12, 13^.

In conclusion, by employing bilayer-hBN as a tunnel barrier in a fully hBN-encapsulated graphene vdW heterostructure, we observe a unique sign inversion and bias induced spin-injection (detection) polarizations between 50% (135%) at +0.6 V and −70% (−60%) at −0.4 V at room temperature. This resulted in a large change in the magnitude of the non-local differential spin signal with the applied DC bias across the Co/2L-hBN/graphene contacts and the inversion of its sign around zero bias. Such a large injection and detection polarizations of the contacts at high bias made it possible to observe the two-terminal differential and DC spin signals reaching up to 800 Ω, and magnetoresistance ratio up to 2.7% even at room temperature. Moreover, we obtain a very large spin accumulation of about 4.1 meV underneath the contacts in a two-terminal spin-valve measurement.

Note that we have been conservative in biasing the contacts to prevent breakdown of the 2L-hBN barriers. By increasing the bias to the maximum theoretical limit of ~±0.8 V^20^, we expect that we can increase the polarizations even further. Also, one can increase the width of the contacts by a factor of 5 to about 1 μm (yet far below *λ*_s_), which will reduce the background resistance of two-terminal spin-valve signal by the same factor, and allow to apply a maximum current bias up to 100 μA^21^. This could result in two-terminal spin signal above 50 mV and MR ratio beyond 20%. The corresponding change in spin accumulation could reach up to 40 meV underneath the contacts, exceeding the room temperature thermal energy (*k*_B_ *T* ~ 25 meV). Such high values of spin accumulation will open up an entirely new regime for studying spin transport in graphene and for applications of graphene based spintronic devices^2^.

## Methods

### Sample preparation

A fully encapsulated hBN/graphene/hBN heterostructure is prepared via a dry pickup transfer method developed in our group^14^. The graphene flake is exfoliated from a bulk HOPG (highly oriented pyrolytic graphite) ZYA grade crystal (SPI) onto a pre-cleaned SiO_2_/Si substrate (${t_{{\rm{Si}}{{\rm{O}}_{\rm{2}}}}}$ = 300 nm). A single layer is identified via the optical contrast analysis. Boron nitride flakes (supplier: HQ Graphene) are exfoliated onto a different SiO_2_/Si substrate (${t_{{\rm{Si}}{{\rm{O}}_{\rm{2}}}}}$ = 90 nm) from small hBN crystals (~1 mm). The thickness of the desired hBN flake is characterized via the Atomic Force Microscopy. For the stack preparation, a bilayer-hBN (2L-hBN) flake on a SiO_2_/Si is brought in contact with a viscoelastic PDMS (polydimethylsiloxane) stamp which has a polycarbonate (PC) film attached to it in a transfer stage arrangement. When the sticky PC film comes in a contact with a 2L-hBN flake, the flake is picked up by the PC film. A single layer graphene (Gr) flake, exfoliated onto a different SiO_2_/Si substrate is aligned with respect to the already picked up 2L-hBN flake in the transfer stage. When the graphene flake is brought in contact with the 2L-hBN flake on the PC film, it is picked up by the 2L-hBN flake due to vdW force between the flakes. In the last step, the 2L-hBN/Gr assembly is aligned on top of a 10 nm thick-hBN flake on another SiO_2_/Si substrate and brought in contact with the flake. The whole assembly is heated at an elevated temperature ~150 °C and the PC film with the 2L-hBN/Gr is released onto the thick-hBN flake. The PC film is dissolved by putting the stack in a chloroform solution for 3 h at room temperature. Then the stack is annealed at 350 °C for 5 h in an Ar-H_2_ environment for removing the polymer residues.

### Device fabrication

The electrodes are patterned via the electron beam lithography on the PMMA (poly(methyl methacrylate))-coated 2L-hBN/Gr/hBN stack. Following the development procedure, which selectively removes the PMMA exposed to the electron beam, 65 nm thick FM cobalt electrodes are deposited on top of the 2L-hBN tunnel barrier for the spin polarized electrodes via electron-beam evaporation. Vacuum pressure is maintained at 1 × 10^−7^ mbar during the deposition. To prevent the oxidation of the cobalt, the ferromagnetic electrodes are covered with a 3 nm thick aluminum layer. The material on top of the unexposed polymer is removed via the lift-off process in hot acetone solution at 50 °C, leaving only the contacts in the desired area.

### Data availability

The data that support the findings of this study are available from the corresponding authors upon request.

## Electronic supplementary material

Supplementary Information
