# Supplementary material for: Bias induced up to 100% spin-injection and detection polarizations in ferromagnet/bilayer-hBN/graphene/hBN heterostructures
Source: Nat Commun. 2017 Aug 15;8:248. doi: 10.1038/s41467-017-00317-w (PMC5557974; doi:10.1038/s41467-017-00317-w)
Supplement: Supplementary file 1 — Supplementary Information [file 41467_2017_317_MOESM1_ESM.pdf]

Description of Supplementary Files

File Name: Supplementary Information
Description: Supplementary Figures, Supplementary Tables, Supplementary Notes and Supplementary References

SUPPLEMENTARY INFORMATION

SUPPLEMENTARY NOTE 1: DEVICE CHARACTERIZATION

An optical microscopy image of the 2L-hBN flake and its AFM thickness measurement is shown in Supplementary Fig. 1. Charge and spin transport measurements in graphene are performed using low-frequency (21 Hz) lock-in measurements. All measurements are performed in vacuum ($\sim 1 \times 10^{-7}$ mbar) at room temperature. In order to eliminate the effect of the contact resistances, the graphene resistivity was characterized using a four-terminal local geometry by applying an AC current between contacts 1-13 and measuring the voltage drop across a pair of contacts in between 1 and 13 (see Fig. 1b of the main text). The square resistance R_{sq} of graphene is consistently found to be $\sim$ 400 Ω for different regions, suggesting that the background doping profile is uniform in the fully encapsulated graphene flake.

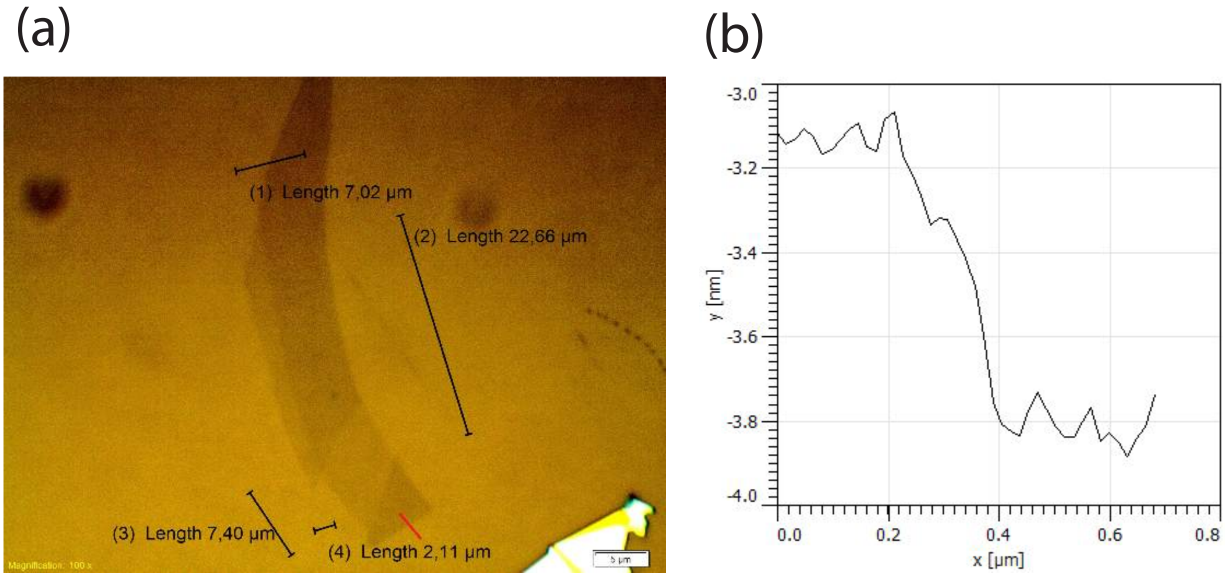

SUPPLEMENTARY FIGURE 1. **hBN tunnel barrier characterization**.(**a**) An optical microscopic image of the hBN tunnel barrier flake on a Si/SiO$_2$ substrate (t_{SiO_2}= 90 nm) where the lighter contrast regions indicate the single-layer hBN. (**b**) An AFM height profile of the 2L-hBN corresponding to the red line drawn in **a**, showing a thickness value $\sim$ 0.7 nm.

The differential contact resistances R_{c}(=dV/dI) of the cobalt/2L-hBN/graphene interface were characterized using a three-terminal connection scheme. For example, to determine the

differential resistance of contact 9, a small and fixed AC current (i) along with a DC current bias (I) is applied between contacts 9-1, and a differential (AC) voltage is measured between 9-13 (Supplementary Fig. 2a) while sweeping the DC bias I. The resulting data is plotted in the Supplementary Fig. 2b. The non-linear behaviour of the high resistive contacts is an indication of the tunneling nature of the 2L-hBN tunnel barrier, whereas the nearly constant differential resistance in the applied bias range, is a characteristic of a transparent (ohmic) contact. For our sample, the differential contact resistances are in the range of 4 - 130 kΩ, and the data is summarized in the Supplementary Table 1.

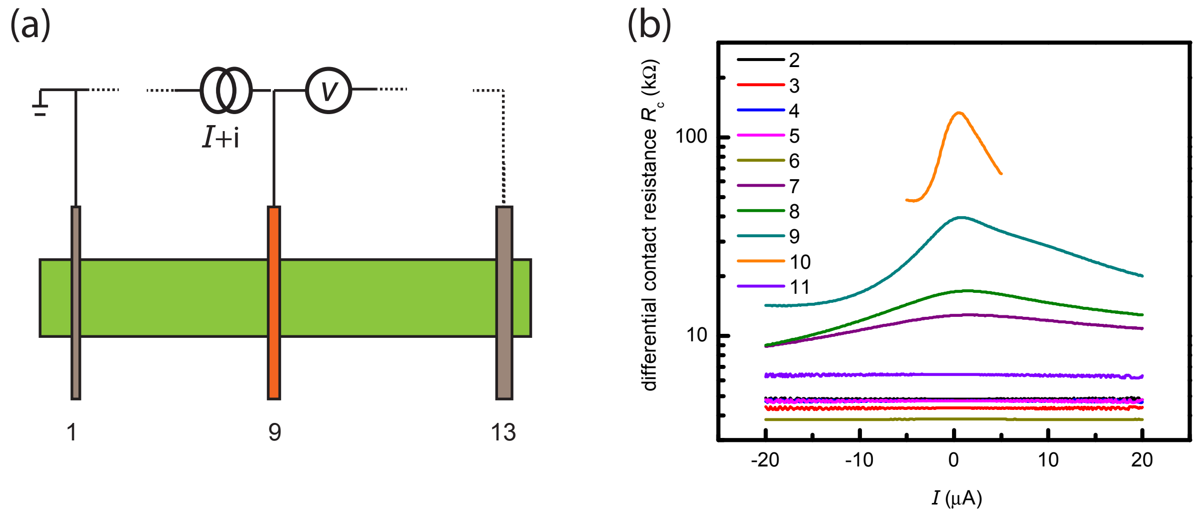

SUPPLEMENTARY FIGURE 2. **Electrical characterization of tunnel barrier**. (**a**) Three-terminal connection scheme for measuring the interface resistance of the cobalt/hBN/graphene contacts (see Supplementary Note 1). (**b**) Differential contact resistance of all the contacts as a function of the DC bias applied across the cobalt/hBN/graphene interfaces.

SUPPLEMENTARY NOTE 2: EXPRESSIONS FOR SPIN-INJECTION AND DETECTION POLARIZATIONS, AND TWO-TERMINAL LOCAL SPIN-SIGNAL

A. Injection polarization

We derive an analytical expression for a DC/AC spin injection and detection polarizations. In our measurements, we observe that the measured polarization depends on the applied DC current bias (I) across the contact. For the DC current injection, the DC

Contact #	R_c (kΩ) (at V=0)	Width of contact (L) (μm)	R_c*Area (kΩ.μm^2)	R_c/R_λ	No. of hBN layers of the barrier
**2**	4.82	0.25	3.61	6.23	1
**3**	4.34	0.20	2.60	5.61	1
**4**	4.74	0.17	2.41	6.12	1
**5**	4.73	0.20	2.83	6.11	1
**6**	3.82	0.40	4.58	4.93	2
**7**	12.7	0.35	13.3	16.4	2
**8**	16.7	0.25	12.5	21.6	2
**9**	38.8	0.15	17.5	50.3	2
**10**	128	0.20	77.1	166	2
**11**	6.41	0.40	7.69	8.28	2
**12**	10.2	0.35	12.2	13.2	2

SUPPLEMENTARY TABLE 1. **A summary of all the used contacts**. Here $R_\lambda = R_{sq}\lambda_s/W = 773\ \Omega$ is the spin resistance of the graphene flake with the width $W = 3\ \mu$m, spin-relaxation length $\lambda_s = 5.8\ \mu$m, and $R_{sq} \sim 400\ \Omega$. The number of hBN layers is determined from the optical contrast analysis of the optical microscopic images and the AFM measurements.

polarization of an injector contact P_{in} is defined as:

$$P_{in}(I) = \frac{I_s}{I} \tag{1}$$

where I_s is the DC spin current and I is the injected DC charge current. Similarly, the AC (differential) polarization of the injection contact p_{in}, in the presence of a DC bias current I, is defined as:

$$p_{in}(I) = \frac{i_s}{i} \tag{2}$$

where i_s is the AC spin current and i is the injected AC charge current.

In our experiment, we apply a DC current at the injector contact along with a small and fixed magnitude of the AC current. The total injected spin current can be represented as:

$$I_s(I + i) = P_{in}(I + i) \times (I + i) \tag{3}$$

Supplementary Eq. 3 can be expanded in to a Taylor series. For a small and fixed AC current i, the second order terms can be neglected and the expression can be rewritten as:

$$I_\mathrm{s}(I) + \left(\frac{\mathrm{d}I_\mathrm{s}}{\mathrm{d}I}\right)\Bigg|_I \times i = P_\mathrm{in}(I) \times I + \left\{P_\mathrm{in}(I) + \left(\frac{\mathrm{d}P_\mathrm{in}}{\mathrm{d}I}\right)\Bigg|_I \times I\right\} \times i \tag{4}$$

The AC (differential) polarization can then be written as:

$$p_\mathrm{in}(I) = \frac{\mathrm{d}I_\mathrm{s}}{\mathrm{d}I} = \frac{i_\mathrm{s}}{i} = P_\mathrm{in}(I) + \left(\frac{\mathrm{d}P_\mathrm{in}}{\mathrm{d}I}\right)\Bigg|_I \times I \tag{5}$$

Supplementary Eq. 5 can be used for a consistency check between the measured p_in and $P_\mathrm{in}(I)$ (Supplementary Fig. 6).

In our case, we observe that p_in approximately scales linearly with bias I, implying that $\frac{\mathrm{d}P_\mathrm{in}}{\mathrm{d}I} \sim$ constant. Supplementary Eq. 5 then gives $P_\mathrm{in} \approx \frac{1}{2} p_\mathrm{in}(I)$.

B. Detection polarization

The spin-detection polarization is defined as a voltage measured at the detector due to the spin accumulation underneath the detector contact. A charge current $\triangle I$ will flow in the ferromagnet via a spin-charge coupling due to a change in the spin accumulation $\triangle\mu_\mathrm{s}$ underneath the detector:

$$\triangle I = \triangle\mu_\mathrm{s}\left(\frac{\mathrm{d}I_\uparrow}{\mathrm{d}V} - \frac{\mathrm{d}I_\downarrow}{\mathrm{d}V}\right) \tag{6}$$

where the net spin accumulation μ_s is the splitting of spin chemical potentials spin-up $\mu_\uparrow$ and spin-down $\mu_\downarrow$, i.e., $(\mu_\uparrow - \mu_\downarrow)/2$. Note that Supplementary Eq. 6 holds under the condition of independent spin channels. For a fixed current bias I at the detector, to compensate for $\triangle I$, the change in the voltage $\triangle V$ at the detector will give rise to a change in the charge current $\triangle I$ in the opposite direction:

$$\triangle I = \triangle V\left(\frac{\mathrm{d}I_\uparrow}{\mathrm{d}V} + \frac{\mathrm{d}I_\downarrow}{\mathrm{d}V}\right) \tag{7}$$

Solving Supplementary equations 6 and 7 leads to:

$$\frac{\triangle V}{\triangle\mu_\mathrm{s}} = \frac{\frac{\mathrm{d}I_\uparrow}{\mathrm{d}V} - \frac{\mathrm{d}I_\downarrow}{\mathrm{d}V}}{\frac{\mathrm{d}I_\uparrow}{\mathrm{d}V} + \frac{\mathrm{d}I_\downarrow}{\mathrm{d}V}} = \frac{\mathrm{d}I_\uparrow - \mathrm{d}I_\downarrow}{\mathrm{d}I_\uparrow + \mathrm{d}I_\downarrow} = \frac{\mathrm{d}I_\mathrm{s}}{\mathrm{d}I} \tag{8}$$

Since the spin accumulations underneath the detector contacts are generally small, this equation is valid for both the DC detector polarization P_d and the differential detector

polarization $p_{\rm d}$, i.e.,

$$P_{\rm d}(I) = p_{\rm d}(I) = \frac{{\rm d}I_{\rm s}}{{\rm d}I} = p_{\rm in}(I) \tag{9}$$

Note that electrons can only inject one spin $\hbar/2$ (up or down), which implies that $P_{\rm in}(I)$ is restricted below $\pm$ 100%. However, this does not hold for the differential injection polarization $p_{\rm in}$ as well as detection polarizations $p_{\rm d}(I)$ and $P_{\rm d}(I)$ which can in principle exceed $\pm$100% in case of applied bias. Note however that when a detector is biased, it will also inject spins resulting in a spin accumulation underneath the detector. When the detector is fully spin polarized, the spin induced voltage V cannot exceed the total spin accumulation $\pm\mu_{\rm s,\,total}/e$ (due to injector and detector). As it can be seen from the Table I of the main text, this condition is always satisfied, since the sum of the spin induced voltages cannot be larger than $\mu_{\rm s,\,total}/e$ = (3.9+4.1)/e = 8 mV which is in agreement with the signal in Figure 5d of the main text.

C. Two-terminal local spin signals

We can calculate the bias-dependent two-terminal spin signal, provided the spin injection and detection polarizations are known. For the two-terminal measurements, the injector and detector are both biased with the same DC current I but they are biased with opposite polarity. The two-terminal DC spin signal $\triangle V_{\rm 2t}^{\rm DC}$ between contacts 8 and 9 (See Fig. 5 in the main text) can be written as:

$$\triangle V_{\rm 2t}^{\rm DC} = I \times [P_{\rm in}^{9}(I)P_{\rm d}^{8}(-I) + P_{\rm in}^{8}(-I)P_{\rm d}^{9}(I)] \times \frac{R_{\rm sq}\lambda_{\rm s}}{W} \times e^{-\frac{L}{\lambda_{\rm s}}} \tag{10}$$

which is equal to $V_{\rm 2t}^{\uparrow\uparrow}(I) - V_{\rm 2t}^{\downarrow\uparrow}(I)$, the difference in two-terminal DC voltage signal $V_{\rm 2t}^{\rm DC}$ when the magnetization configuration of contacts 8 and 9 changes between parallel($\uparrow\uparrow$) and anti-parallel($\downarrow\uparrow$) (see the main text).

Similarly, the two-terminal differential spin signal $\triangle R_{\rm 2t}^{\rm AC}$ between contacts 8 and 9 (See Fig. 5 in the main text) can be written as:

$$\triangle R_{\rm 2t}^{\rm AC} = [p_{\rm in}^{9}(I)p_{\rm d}^{8}(-I) + p_{\rm in}^{8}(-I)p_{\rm d}^{9}(I)] \times \frac{R_{\rm sq}\lambda_{\rm s}}{W} \times e^{-\frac{L}{\lambda_{\rm s}}} \tag{11}$$

which is equal to $R_{\rm 2t}^{\uparrow\uparrow}(I) - R_{\rm 2t}^{\downarrow\uparrow}(I)$, the difference in the two-terminal differential signal $R_{\rm 2t}^{\rm AC}$ when the magnetization configuration of contacts 8 and 9 changes between parallel($\uparrow\uparrow$) and anti-parallel($\downarrow\uparrow$).

Here L is the separation between the contacts 8 and 9. $p_{\rm in}$ and $p_{\rm d}$ are obtained by following the procedure explained in the Supplementary Notes 3.

SUPPLEMENTARY NOTE 3: DETERMINING THE BIAS DEPENDENT SPIN-INJECTION POLARIZATIONS FROM NON-LOCAL SPIN SIGNALS

In a typical non-local spin-valve measurement, a differential voltage signal $v_{\rm nl}$, measured by a detector contact 'd' with differential detection polarization $p_{\rm d}$, located at a distance L from an injector contact 'in' with differential injection polarization $p_{\rm in}$, is given by

$$v_{\rm nl} = \frac{iR_{\rm sq}\lambda_{\rm s}}{2W} p_{\rm in} p_{\rm d} e^{-L/\lambda_{\rm s}} \tag{12}$$

where $R_{\rm sq}$ is the square resistance of graphene, $\lambda_{\rm s}$ is the spin relaxation length in graphene and W is the width of the graphene flake.

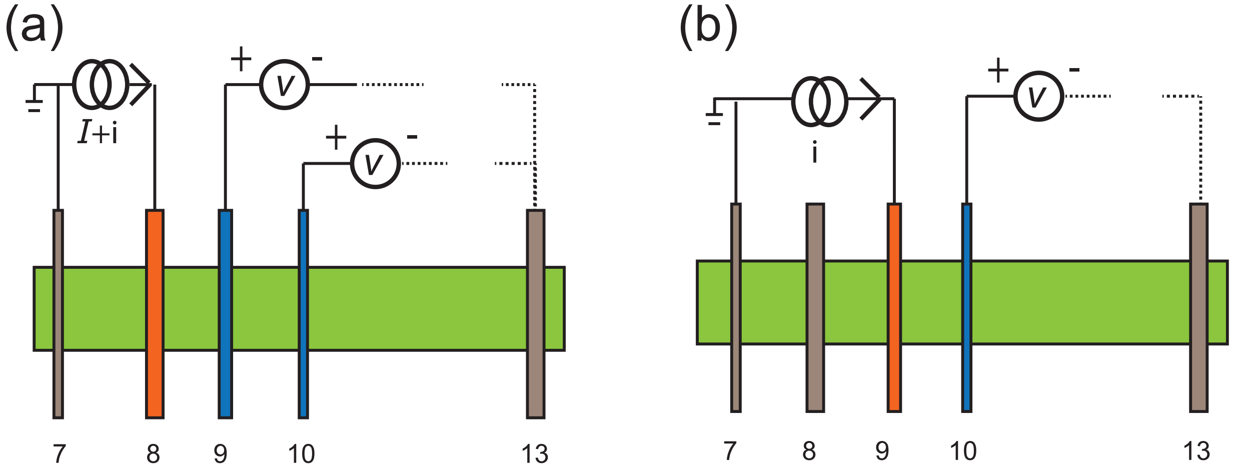

SUPPLEMENTARY FIGURE 3. **Schematics of the measurement configurations for determining the spin-injection polarization of the contact 8**. (**a**) Measuring non-local spin signals as a function of bias on injector contact. (**b**) Measuring unbiased non-local spin signal with the two detector contacts 9 and 10.

Consider a group of five contacts 7, 8, 9, 10, and 13 in Supplementary Fig. 3a, where the current $(I + i)$ is injected through a ferromagnet in contact 8 and extracted through 7, and the total differential spin accumulation is detected as a non-local differential voltage, using a low-frequency lock-in detection scheme, between the contacts 9 and 13 $v_{\rm nl}^{9-13}$.

The non-local voltage measured with the magnetization of the contacts 7, 8, 9, and 13 are aligned in one direction (say ↑↑↑↑) is given by,

$$v_{\mathrm{nl}}^{9-13}(\uparrow\uparrow\uparrow\uparrow) = \frac{iR_{\mathrm{sq}}\lambda_s}{2W}\left[p_9\left(p_8 e^{-L_{8-9}/\lambda_{\mathrm{s}}} - p_7 e^{-L_{7-9}/\lambda_{\mathrm{s}}}\right) - p_{13}\left(p_8 e^{-L_{8-13}/\lambda_{\mathrm{s}}} - p_7 e^{-L_{7-13}/\lambda_{\mathrm{s}}}\right)\right] \tag{13}$$

In our measurements, the outer detector 13 is far enough from the injectors (L_{7-13}, L_{8-13} $>$ 2-3*λ_{s}) to not detect any spin signal and serves as a reference detector for the rest of the analysis. So, the non-local differential resistance $R_{\mathrm{nl}} = v_{\mathrm{nl}}/i$ detected by 9 due to injection from 7 and 8 is given by

$$R_{\mathrm{nl}}^{\uparrow\uparrow\uparrow} = \frac{R_{\mathrm{sq}}\lambda_{\mathrm{s}}}{2W}\left[p_9\left(p_8 e^{-L_{8-9}/\lambda_{\mathrm{s}}} - p_7 e^{-L_{7-9}/\lambda_{\mathrm{s}}}\right)\right] \tag{14}$$

In a spin-valve measurement, when the magnetization of one of the contact (say, 8) switches, the resulting non-local resistance can be written as,

$$R_{\mathrm{nl}}^{\uparrow\downarrow\uparrow} = \frac{R_{\mathrm{sq}}\lambda_{\mathrm{s}}}{2W}\left[p_9\left(-p_8 e^{-L_{8-9}/\lambda_{\mathrm{s}}} - p_7 e^{-L_{7-9}/\lambda_{\mathrm{s}}}\right)\right] \tag{15}$$

The detected signals in the Supplementary equations 14 and 15 include the contribution of spin signal from the outer injector 7 (second term of the expressions) as well as some field independent background signal.

Since the only change in Supplementary equations 14 and 15 is due to contact 8, the non-local spin signal measured by 9 corresponding to the spin accumulation created only by 8 is obtained from

$$\Delta R_{\mathrm{nl}}^{8-9} = \frac{R_{\mathrm{nl}}^{\uparrow\uparrow\uparrow} - R_{\mathrm{nl}}^{\uparrow\downarrow\uparrow}}{2} = \frac{R_{\mathrm{sq}}\lambda_{\mathrm{s}}}{2W}\left[p_9\left(p_8 e^{\frac{-L_{8-9}}{\lambda_{\mathrm{s}}}}\right)\right] \tag{16}$$

As explained above, one can determine the spin signal measured via inner detector contact 9 correspond to the spin injection through inner injector contact 8 as given by Supplementary Eq. 16. Further, as shown in Supplementary Fig. 3(b), we can simultaneously measure the spin signal via inner detector contact 10 corresponding to the spin injection through inner injector contact 8, given by

$$\Delta R_{\mathrm{nl}}^{8-10} = \frac{R_{\mathrm{nl}}^{\uparrow\uparrow\uparrow} - R_{\mathrm{nl}}^{\uparrow\downarrow\uparrow}}{2} = \frac{R_{\mathrm{sq}}\lambda_{\mathrm{s}}}{2W}\left[p_{10}\left(p_8 e^{\frac{-L_{8-10}}{\lambda_{\mathrm{s}}}}\right)\right] \tag{17}$$

The contact polarization of the contacts 9 and 10 can be expressed as a ratio of $\triangle R_{\rm nl}^{8\text{-}9}$ and $\triangle R_{\rm nl}^{8\text{-}10}$ i.e.

$$\frac{p_9}{p_{10}} = \frac{\triangle R_{\rm nl}^{8\text{-}9}}{\triangle R_{\rm nl}^{8\text{-}10}} e^{\frac{-L_{9-10}}{\lambda_{\rm s}}} \tag{18}$$

In order to determine the unbiased values of detector polarizations p_9 and p_{10}, we need one more equation with these variables which is obtained by measuring $\triangle R_{\rm nl}$ between 9 and 10, by applying only an AC injection current between contacts 7 and 9 and measuring a non-local voltage between 10 and 13. The effect of the outer injector contact 7 is subtracted using the procedure described above(see Supplementary equations 13 - 16). Now we obtain:

$$\Delta R_{\rm nl}^{9-10} = \frac{R_{\rm sq}\lambda_{\rm s}}{2W}\left[p_{10}\left(p_9 e^{\frac{-L_{9-10}}{\lambda_{\rm s}}}\right)\right] \tag{19}$$

We can obtain the product $p_9 \times p_{10}$ from Supplementary Eq. 19 and the ratio $\frac{p_9}{p_{10}}$ from Supplementary Eq. 18 and thus determine the unbiased polarizations p_9 and p_{10}.

Using the unbiased polarization values of detectors obtained from Supplementary equations 18 and 19, we can determine the bias dependent polarization of the injector contact 8 from the two non-local spin signals measured via contacts 9 (Supplementary Eq. 16) and contact 10 (Supplementary Eq. 17), independently. The resulting differential spin-injection polarization of contact 8 is plotted in Supplementary Fig. 4(b).

The above procedure is repeated with three more different groups of contacts to determine the differential polarization of injection contacts, and the results are plotted in the Supplementary Fig. 4(a-d). The results are also summarized in the Supplementary Table 2.

SUPPLEMENTARY NOTE 4: DETERMINING THE BIAS-DEPENDENT DETECTOR POLARIZATIONS

In order to measure the bias dependent detector polarization of contact 9, we keep the injector contact 8 at a fixed DC current bias I, where $p_8(I)$ is known from the previous measurements, and sweep a bias current $I_{\rm d}$ across the detector 9. We apply a fixed I and a small i through the injector electrode 8 and measure a non-local signal at detector 9 via low-frequency lock-in detection method, while sweeping the DC current bias $I_{\rm d}$ across the detector 9 (Supplementary Fig. 5). Note that the spin transport is non-local only for the AC measurements. For the DC measurements we have a non-zero charge current and an electric field in the spin transport channel between contacts 8 and 9. A differential non-local

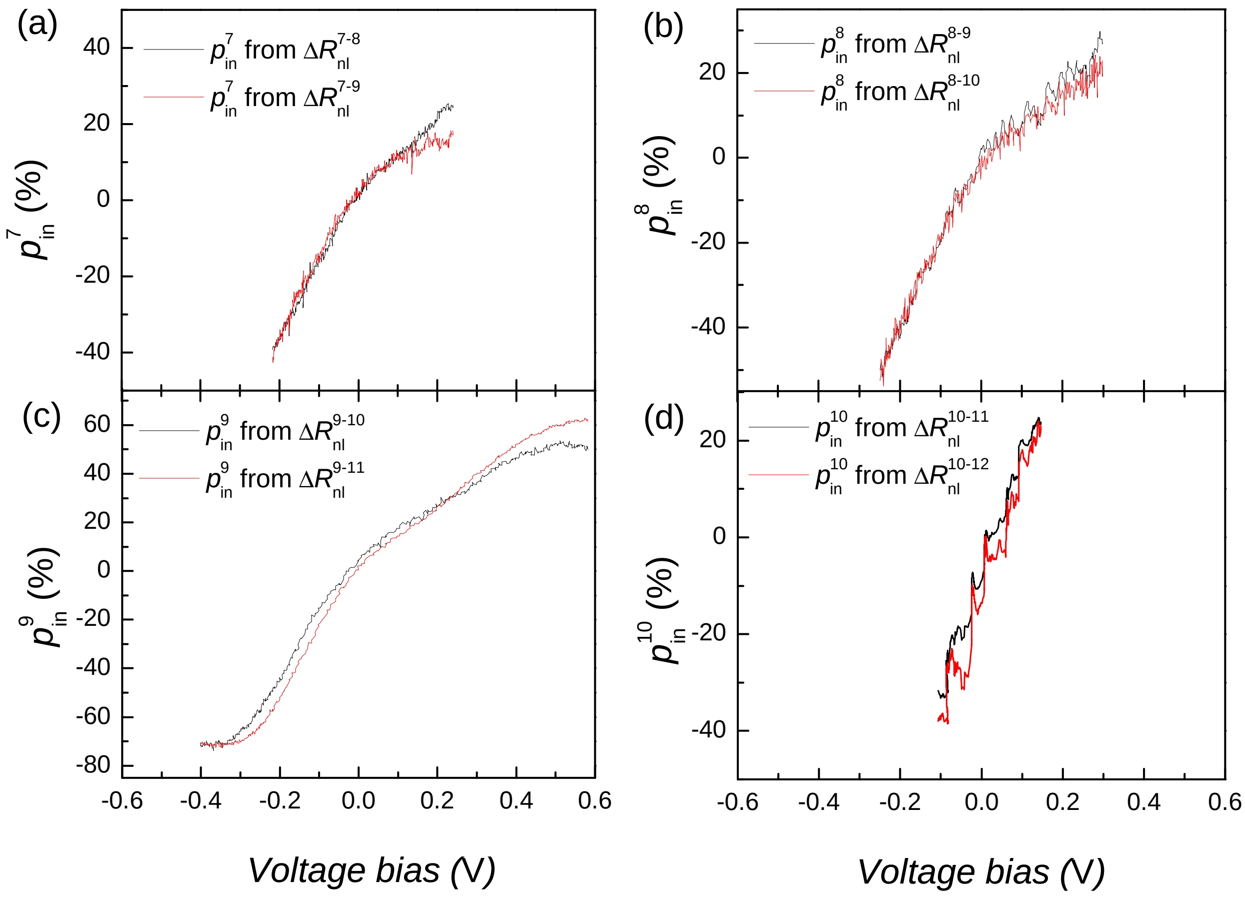

SUPPLEMENTARY FIGURE 4. **Differential spin-injection polarization for different injector contacts as a function of DC voltage bias across the injector**. The spin injection into graphene from the FM cobalt is facilitated via a 2L-hBN tunnel barrier, clearly demonstrating the change in the magnitude and the sign of the injector polarization as a function of the bias current I . The injection polarizations of contact 7 p_7 in (**a**), contact 8 $p_8$in (**b**), contact 9 p_9 in (**c**), and contact 10 p_{10} in (**d**) are shown.

signal $\triangle R_{\rm nl}^{8\text{-}9}$ is measured as a function of detector bias current $I_{\rm d}$ and can be expressed via Supplementary Eq. 16. Here, we know the spin-injection polarization $p_8(I)$ obtained from the previous measurements (Supplementary Note 3) and can extract the spin detection polarization as a function of the bias current $I_{\rm d}$ using Supplementary Eq. 16 (see the main text).

Set of contacts	Injector-detector $(in - d)$	At V = 0: p_{in} (%)	At V = 0: p_{d} (%)	At V = +V$_{max}$: $\Delta R_{\text{nl}}^{in-d}$ (Ω)	At V = +V$_{max}$: p_{in} (%)	At V = -V$_{max}$: $\Delta R_{\text{nl}}^{in-d}$ (Ω)	At V = -V$_{max}$: p_{in} (%)
7-8-9	7-8	p_7 = 1.4	p_8 = -2.0	-1.5	24.5	2.3	-38.5
	7-9		p_9 = 1.1	0.5	17.3	-1.1	-42.6
8-9-10	8-9	p_8 = -2.3	p_9 = 1.3	1.2	26.9	-1.9	-50.0
	8-10		p_{10} = 3.0	1.6	22.9	-3.8	-52.6
9-10-11	9-10	p_9 = 4.3	p_{10} = 2.4	3.7	51.3	-5.2	-71.0
	9-11		p_{11} = 3.2	3.9	61.8	-4.5	-70.8
10-11-12	10-11	p_{10} = -1.7	p_{11} = 3.2	1.9	23.2	-2.6	-31.6
	10-12		p_{12} = 2.0	0.9	23.1	-1.5	-37.9

SUPPLEMENTARY TABLE 2. **A summary of spin-valve signals and obtained differential spin-injection/detection polarizations**. $\Delta R_{\text{nl}}^{in-d}(V)$ is the non-local signal from spin-valve data when the injection bias V applied across the injector(in) and measured via detector(d), $p_{\text{in}}(V)$ is the differential injection polarization of injector contact at bias V, calculated from the analysis explained in the Supplementary Notes 4, and $V_{max(min)}$ is the maximum(minimum) bias applied across the injector. Here, the detector polarization p_{d} at zero bias obtained from following the analysis described in the Supplementary Note 3.

SUPPLEMENTARY NOTE 5: DIFFERENTIAL POLARIZATION FROM DC POLARIZATION

The differential spin-injection polarization $p_{\text{in}}(I)$ can be expressed as the sum of DC injection polarization $P_{\text{in}}(I)$, and $\left(\frac{\mathrm{d}P_{\text{in}}(I)}{\mathrm{d}I}\right)\Big|_I I$ (Supplementary Eq. 5). We determine the differential spin-injection polarization of contact 8 $p_{\text{in}}^{8}(I)$ as explained in the Supplementary Note 3. A similar analysis is used to determine the DC spin-injection polarization of

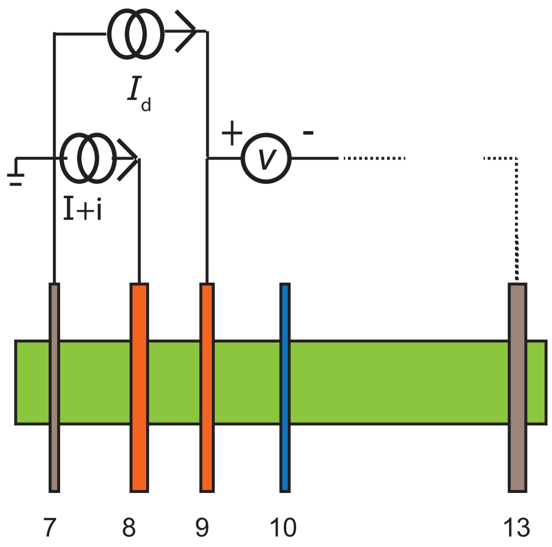

SUPPLEMENTARY FIGURE 5. **Measurement geometry for biasing the detector to measure the spin-detection polarization**

contact 8 $P_{\text{in}}^{8}(I)$ from the DC spin transport measurements where a non-local spin signal is measured via a DC voltmeter. Supplementary Fig. 6 shows $p_{\text{in}}^{8}(I)$ determined both from the measurements and from the analytical expression Supplementary Eq. 5. The measured and the calculated differential polarization ($p_{\text{in}}(I)$) are in a good agreement, supporting the consistency of our approach.

SUPPLEMENTARY NOTE 6: LOW INTERFACE RESISTANCE CONTACTS

As indicated in the optical microscope picture in Fig. 1b of the main text and Supplementary Fig. 1a, a part of the hBN tunnel barrier flake consists of a monolayer(1L)-hBN region. The contacts from 2 to 5, either fully or partially deposited on top of the monolayer region of the tunnel barrier flake, show low interface resistance of $\approx$ 4-5 kΩ, whose differential interface resistance R_{c} ($= \text{d}V/\text{d}I$) is constant as a function of bias (Supplementary Fig. 2b).

Supplementary Figure 7 shows the non-local spin-signal corresponding to the spin injection through the low R_{c} contacts 2 and 4, as a function of the applied bias. For a comparison, the spin signal for the high R_{c} contact 9, $\Delta R_{\text{nl}}^{\text{9-10}}$ is also shown. For the same range of the applied voltage bias, low R_{c} contacts with 1L-hBN tunnel barriers do not show significant change in the spin signal as well as no sign reversal around zero bias. Whereas the high

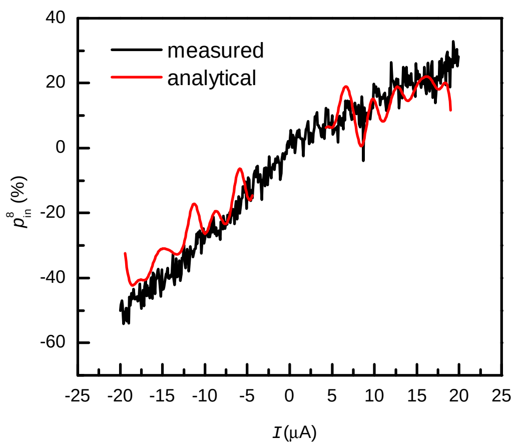

SUPPLEMENTARY FIGURE 6. **Differential spin injection polarization obtained from AC and DC spin transport measurements**.Differential spin-injection polarization of contact 8 obtained from the measurements (black curve) and from the analytical expression Supplementary Eq. 5 (red curve).

resistive contacts, for example 9, with 2L-hBN tunnel barriers, show a large modulation as well as change in sign of the non-local spin-signal.

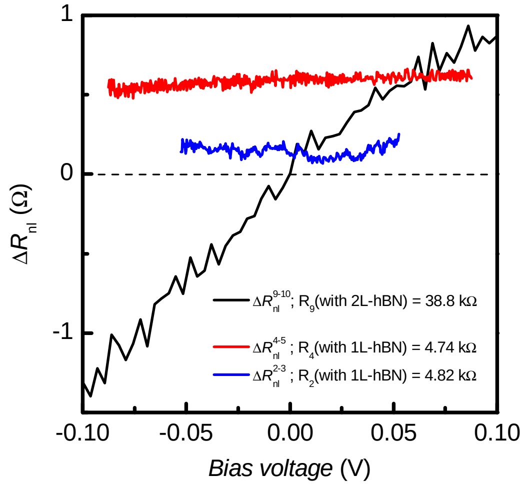

SUPPLEMENTARY FIGURE 7. **Non-local spin signal with mono and bi-layer of hBN barrier**.Comparison of spin signals from low and high resistive contacts with 1L-hBN and 2L-hBN barriers.

For the used contacts $R_c/R_\lambda > 5$ (see Supplementary Table 1) and $L/\lambda \approx 1$, the maximum

reduction in τ_s due to the contact induced spin relaxation is within 10%[1]. Here, $R_\lambda = R_{sq}\lambda_s/W$ with square resistance $R_{sq} \sim 400\ \Omega$, width W = 3 μm of graphene, and spin-relaxation length $\lambda_s = 5.8\ \mu$m.

SUPPLEMENTARY NOTE 7: SPIN-INJECTION DUE TO HEATING

We use a large value of DC current up to $\pm 20\ \mu$A, in order to modulate the spin-injection and -detection polarizations of contacts, which might raise the electron temperature underneath significantly and could inject spins into graphene via a spin-dependent Seebeck effect[2]. We can roughly estimate the electron temperature in graphene due to Joule heating ($VI \sim 10\ \mu$W) at the interface, provided the hBN-SiO_2 thermal resistance (R_{th}) is known.

Since the thermal conductivity of hBN ($\kappa \sim 380$ $Wm^{-1}K$) is 200 times higher than SiO_2 ($\kappa \sim 1.2$ $Wm^{-1}K$), the heat flow will be limited by the SiO_2 thermal conductivity. The effective contact area is about 1 μm^2 and in this area, the heat will flow and spread approximately 1 μm in the SiO_2/Si reservoir. The effective thermal resistance R_{th} of the reservoir will be approximately 3 $\times 10^5$ KW^{-1}. An increase in the temperature $\triangle T$ due to heating can be related as:

$$\triangle T = QR_{th} \tag{20}$$

where Q is the heat transport rate i.e., heating at the interface. We obtain $\triangle T \sim 3$ K on SiO_2/Si substrate.

The high value of the DC current will heat up the tunnel junction and could mimic a spin accumulation due to temperature gradient and the spin dependent Seebeck coefficient of the interface[2]. In our experiments, however, we also demonstrate the modification of the spin-detection polarization along with the spin-injection polarization, which cannot be explained via these effects. Therefore, the effect of heating on the spin transport can be disregarded in our case.

SUPPLEMENTARY NOTE 8: CARRIER DENSITY ESTIMATION UNDERNEATH THE CONTACT

In graphene, the carrier density can be estimated from the Einstein relation:

$$\sigma = \frac{1}{R_{\text{sq}}} = e^2 D_{\text{c}} \nu(E_{\text{F}}) \tag{21}$$

where D_{c} is the charge diffusion coefficient, $\nu(E_{\text{F}})$ is the density of states at the Fermi energy E_{F}, which given by the following equation:

$$\nu(E) = \frac{g_{\text{s}} g_{\text{v}} 2\pi |E|}{h^2 v_{\text{F}}^2} \tag{22}$$

where $g_{\text{s}} = 2$ and g_{v}=2, are the spin degeneracy and the valley degeneracy of the electron, respectively, and $v_{\text{F}} = 10^6\ m/s$, is the Fermi velocity of the electron. The density of the carriers n can be estimated by integrating Supplementary Eq. 22 from zero to E_{F}:

$$n = \frac{g_{\text{s}} g_{\text{v}} \pi E_{\text{F}}^2}{h^2 v_{\text{F}}^2} \tag{23}$$

Using Supplementary equations 22, 23, and 21, n can be obtained from[3]:

$$n = \left(\frac{h v_{\text{F}}}{R_{\text{sq}} 2 e^2 \sqrt{g_{\text{s}} g_{\text{v}}} \sqrt{\pi} D_{\text{c}}} \right)^2 \tag{24}$$

For our device, we measure $R_{\text{sq}} \sim 400\ \Omega$. In the absence of the magnetic moments, the charge (D_{c}) and the spin spin diffusion coefficient (D_{s}) will be equal[4]. From the spin transport measurements, we extract $D_{\text{s}} = 0.04\ m^2/s$ and use this value to estimate n in the graphene flake from Supplementary Eq. 24 $\sim$ 5*10^{12} cm^{-2}. Using the relation $\sigma = ne\mu$, we estimate the carrier mobility $\mu \sim 3000$ cm^{2}V^{-1}s^{-1}.

When a bias is applied across a cobalt/2L-hBN tunnel barrier, it modifies the carrier density underneath the contact[5]. In order to estimate this, we assume that initially, the graphene is undoped ($E_{\text{F}} = 0$) underneath the contact. However, the actual doping is unknown. On applying the bias V, the Fermi level is changed by $\triangle E_{\text{F}}$:

$$\triangle n = \frac{g_{\text{s}} g_{\text{v}} \pi \triangle E_{\text{F}}^2}{h^2 v_{\text{F}}^2} \tag{25}$$

which can be related with the external bias V with the following relation:

$$\triangle n = C_{\text{o}}(V - \frac{\triangle E_{\text{F}}}{e}) = \frac{\epsilon_0 \epsilon_r}{\text{d}}(V - \frac{\triangle E_{\text{F}}}{e}) \tag{26}$$

Here, C_{o} is the geometrical capacitance of the 2L-hBN tunnel barrier, ϵ_0 is the dielectric permittivity (= 8.85×10^{-12} F/m), ϵ_r is the relative dielectric permittivity of the hBN ($\sim$

4), e is the electronic charge, and d is the thickness of the tunnel barrier ($= 7\,\text{Å}$). Now, we can obtain $\triangle E_{\mathrm{F}}$ by combining Supplementary Eq. 25 and 26:

$$\triangle E_{\mathrm{F}} = \frac{\pm\sqrt{1+4ceV}-1}{2c} \tag{27}$$

where $c = (4\pi de^2)/(h^2 v_{\mathrm{F}}^2 \epsilon_0 \epsilon_r)$

We obtain $\triangle E_{\mathrm{F}}$ and $\triangle n$ from the equations 27 and 25. For the applied bias $V \sim \pm$ 0.6 V across the tunnel barrier, n can be modified up to $\pm$ 8*10^{12} cm^{-2}, implying that it is possible to tune the carrier density underneath the contact from p- to n-type or vice versa around the charge neutrality point.

SUPPLEMENTARY NOTE 9: DRIFT EFFECTS ON SPIN INJECTION/DETECTION POLARIZATION AND SPIN TRANSPORT

Jozsa et al.[6] reported an enhanced differential spin-injection polarization using the pinhole Al_2O_3 barriers from 18% at zero DC current bias upto 31% at +5 μA bias, while it approaches zero at reverse bias due to a strong local carrier drift near the low resistive regions beneath the contact. On the contrary, we observe an increase in the magnitude of the differential polarization and a change in the sign on reversing the bias. This indicates that the observed behaviour in our device is not due to the carrier drift.

The presence of a non-zero electric-field in the graphene spin transport channel could also modify λ_{s}. The spin relaxation length due to the positive drift field (upstream of spins)λ_+, and due to the negative drift field (downstream of spins) λ_- can be calculated from[7]

$$\frac{1}{\lambda_\pm} = \pm\frac{v_{\mathrm{d}}}{2D_{\mathrm{s}}} + \sqrt{\left(\frac{1}{\sqrt{\tau_{\mathrm{s}}D_{\mathrm{s}}}}\right)^2 + \left(\frac{v_{\mathrm{d}}}{2D_{\mathrm{s}}}\right)^2} \tag{28}$$

Here $v_{\mathrm{d}} = \mu E$ is the drift velocity of the electron(or hole) in an electric-field $E = IR_{\mathrm{sq}}/L$, μ is the field-effect carrier mobility, and L is length of the spin-transport channel. For an applied bias of 20 μA and channel length of 1 μm with a carrier mobility $\sim$ 3000 cm^{2}V^{-1}s^{-1}, the calculations lead to $\lambda_+ = 4.9$ μm and $\lambda_- = 6.7$ μm, whereas the spin relaxation length obtained from the Hanle fitting, under zero bias, is 5.8 μm which is nearly equal to the average of λ_+ and λ_-. The polarization values, obtained using λ_+ or λ_-, differ by 10%, compared to that extracted using λ_{s} in the absence of the drift field. This implies that the injector and detector polarizations also have a similar uncertainty.

SUPPLEMENTARY NOTE 10: BIAS DEPENDENCE FOR CO/$\mathbf{TIO_2}$/GRAPHENE TUNNELING CONTACTS

We also perform the same experiment on a reference sample with TiO_2 tunnel barriers. The contact resistance for FM electrodes with the TiO_2 was around 40 kΩ which is comparable to the interface resistance of the contacts with a 2L-hBN tunnel barrier. However, we do not see any sign reversal of the non-local spin-signal ($\triangle R_{\mathrm{nl}}$) within the range of applied bias I on injector contact. Also, the magnitude of $\triangle R_{\mathrm{nl}}$ is hardly modified (Supplementary Fig. 8).

SUPPLEMENTARY NOTE 11: ADDITIONAL TWO-TERMINAL SPIN VALVES

Here, we show additional results of two-terminal differential spin valve signals for contact configurations with a contact separation of 1.5 m and 2.5 m. The two-terminal spin valve measurement configuration is depicted in the inset of Fig. 5 of the main text.

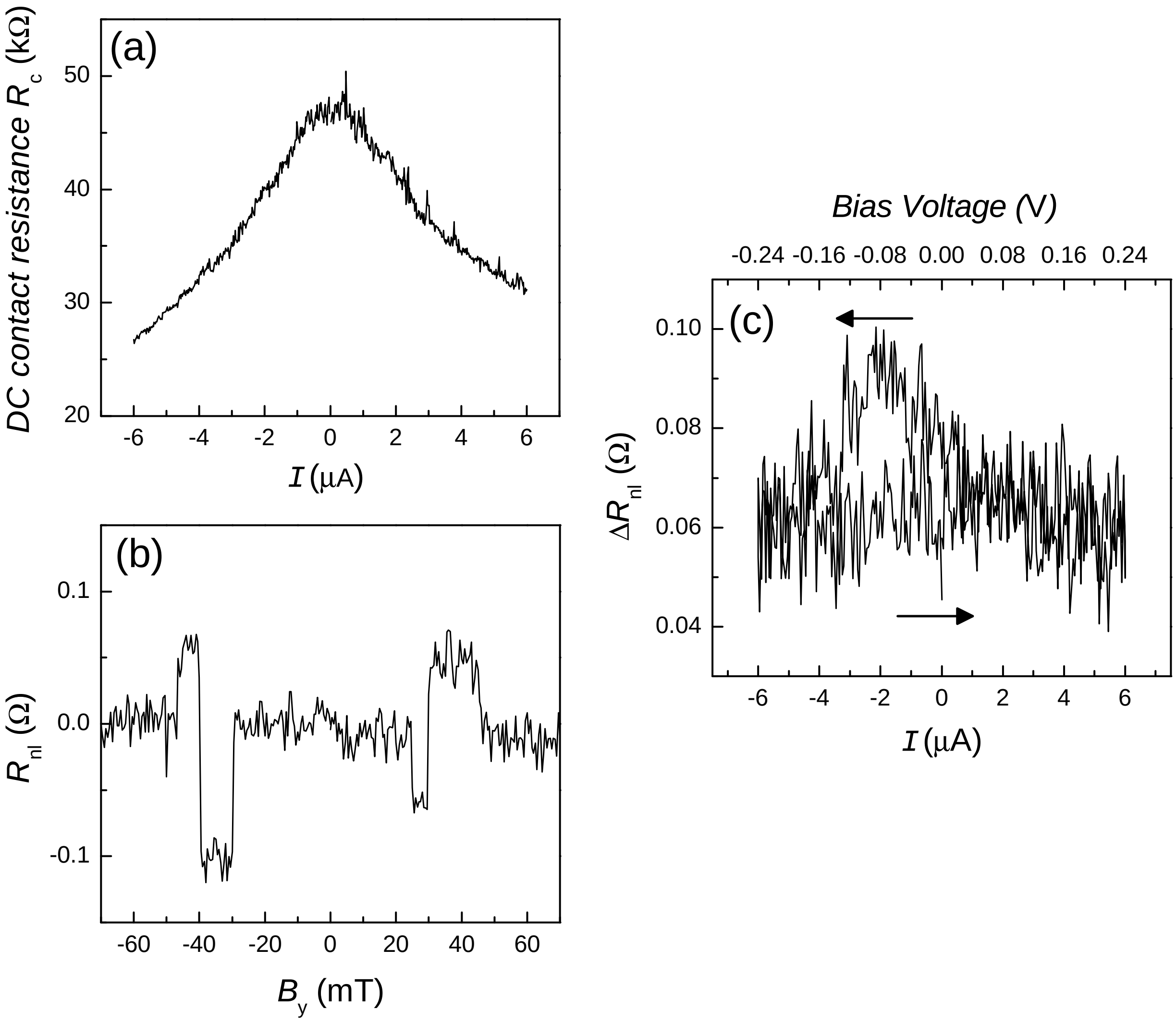

SUPPLEMENTARY FIGURE 8. (**Bias dependent spin signal with oxide tunnel barrier**. (**a**) DC contact resistance $R_c(= V/I)$ of a Co/TiO_2/graphene tunnel barrier shows a non-linear behaviour as a function of DC current bias I, implying a tunneling behaviour of contacts. (**b**) A spin-valve measurement for graphene with TiO_2 tunnel barriers. An offset at zero field is subtracted from the non-local resistance. (**c**) Non-local spin signal $\triangle R_{\rm nl}$ for the spin injection through an injector electrode with TiO_2 tunnel barrier a function of DC current bias. Arrows indicate the direction of the bias sweep. In contrast to the contacts with 2L-hBN tunnel barriers (Supplementary Fig. 7), the contacts with TiO_2 barriers show no change in the magnitude and the sign of the injection polarization as a function of I.

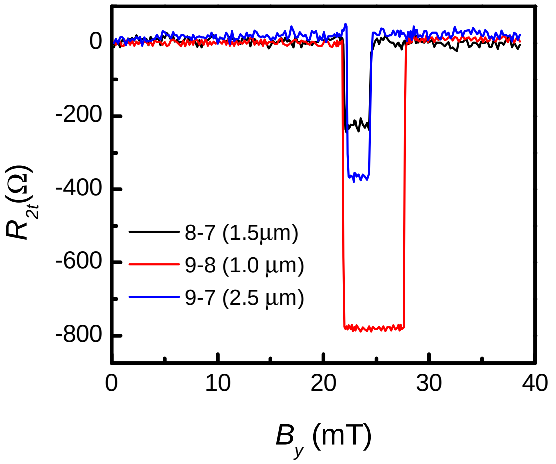

SUPPLEMENTARY FIGURE 9. **Additional two-terminal spin valve signals**. Two-terminal differential spin-valve signal R_{2t} as a function of $\mathbf{B}_y$ at DC current bias of $+20\ \mu$A for three different pairs of contacts. An offset resistance at zero magnetic field is subtracted from each spin valve data for a clear data representation. The legend indicates the pairs of contacts involved and the contact separations.

SUPPLEMENTARY REFERENCES

[1] Maassen, T., Vera-Marun, I. J., Guimarães, M. H. D. & van Wees, B. J. Contact-induced spin relaxation in hanle spin precession measurements. *Phys. Rev. B* **86**, 235408 (2012).

[2] Vera-Marun, I., van Wees, B. & Jansen, R. Spin Heat Accumulation Induced by Tunneling from a Ferromagnet. *Phys. Rev. Lett.* **112**, 056602 (2014).

[3] Józsa, C. *et al.* Linear scaling between momentum and spin scattering in graphene. *Phys. Rev. B* **80**, 241403 (2009).

[4] Weber, C. P. *et al.* Observation of spin Coulomb drag in a two-dimensional electron gas. *Nature* **437**, 1330–1333 (2005).

[5] Bokdam, M., Khomyakov, P. A., Brocks, G., Zhong, Z. & Kelly, P. J. Electrostatic Doping of Graphene through Ultrathin Hexagonal Boron Nitride Films. *Nano Lett.* **11**, 4631–4635 (2011).

[6] Józsa, C., Popinciuc, M., Tombros, N., Jonkman, H. T. & van Wees, B. J. Controlling the efficiency of spin injection into graphene by carrier drift. *Phys. Rev. B* **79**, 081402 (2009).

[7] Ingla-Aynés, J., Meijerink, R. J. & van Wees, B. J. Eighty-Eight Percent Directional Guiding of Spin Currents with 90 μm Relaxation Length in Bilayer Graphene Using Carrier Drift. *Nano*

Lett. **16**, 4825–4830 (2016).
